# Fibrosing mediastinitis: when to suspect and how to evaluate?

**DOI:** 10.1259/bjrcr.20150274

**Published:** 2016-01-19

**Authors:** Neeraj Jain, Udit Chauhan, Sunil Kumar Puri, Sachin Agrawal, Lalit Garg

**Affiliations:** Department of Radiodiagnosis, G B Institute of Postgraduate Medical Education and Research, New Delhi, India

## Abstract

Fibrosing mediastinitis (FM), which is also known as mediastinal fibrosis or sclerosing mediastinitis, is an uncommon, benign and progressive condition characterized by an invasive proliferation of fibrous tissue within the mediastinum. Tuberculosis and histoplasmosis are the major causes of the granulomatous variety, while non-granulomatous FM is an idiopathic reaction to autoimmune syndromes, drugs and radiation. Contrast-enhanced CT is the investigation of choice that can diagnose, and assess the extent and the severity of involvement. We are presenting a case of FM in a young female who presented with complaints of breathlessness, occasional cough and diffuse chest pain for 3 months.

## Case summary

A 22-year-old female presented with gradually worsening breathlessness, occasional cough and diffuse chest pain for 3 months. There was no history of fever, haemoptysis or palpitation. Various investigations, including an electrocardiogram, echocardiography, Mantoux and routine blood investigations, were unremarkable. A chest X-ray revealed superior mediastinal widening. A contrast-enhanced CT (CECT) scan of the chest was performed for further evaluation of cause of breathlessness and characterization of chest radiograph findings.

## Imaging findings

**Figure 1.**
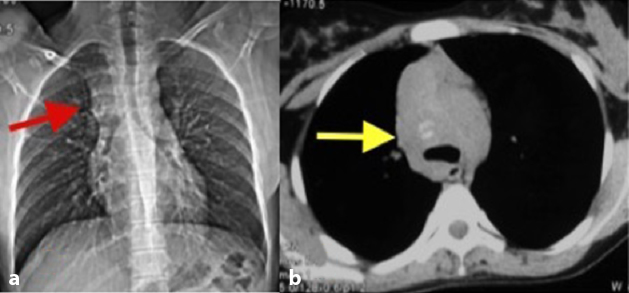
Chest radiograph (a) reveals widening of the superior mediastinum (arrow) and an axial non-contrast CT scan of the chest (b) in the mediastinal window shows an ill-defined mass with faint amorphous calcification (arrow).

**Figure 2.**
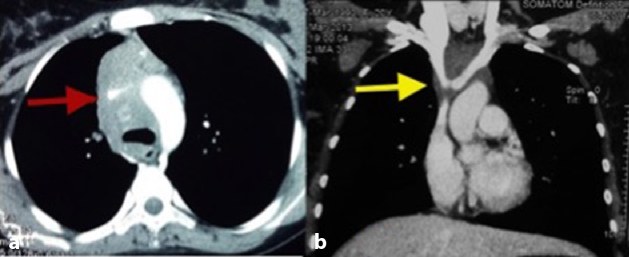
A contrast-enhanced CT scan of the chest. Axial image in the mediastinal window (a) shows an ill-defined, hypodense mass causing attenuated calibre of the superior vena cava (arrow) and narrowing of the proximal segments of the brachiocephalic veins (arrow) in the coronal image (b).

**Figure 3.**
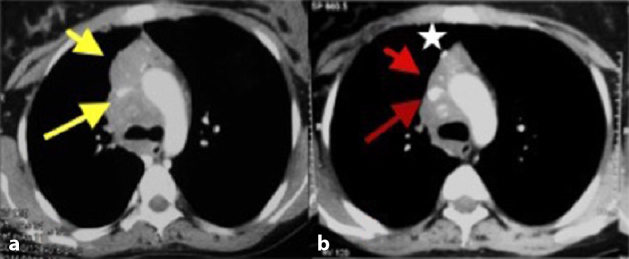
Pre-treatment (a) and post-treatment (1 year; b) axial post-contrast CT images showing significant interval regression of the mass (short arrows in a and b) and superior vena cava luminal compromise (long arrows in a and b) following treatment. The post-biopsy metallic clip is noteworthy (star in b).

**Figure 4.**
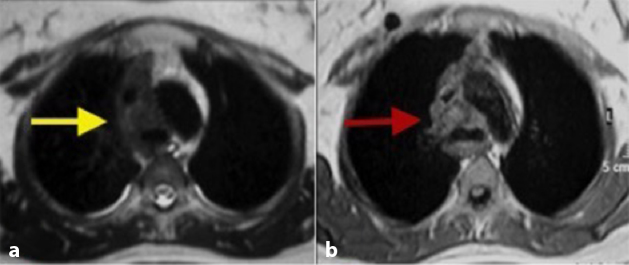
Axial *T*_2_ (a) and *T*_1 _weighted (b) images of the mediastinum depicting the hypointense nature of the mediastinal mass (arrows in a, b) in contrast to the hyperintense normal mediastinal fat.

The plain chest radiograph showed widening of the superior mediastinum as the lone significant finding (Figure 1). The CT scan revealed an ill-defined, hypodense, non-enhancing soft-tissue attenuation lesion with subtle amorphous calcification involving the superior mediastinum, encasing the major mediastinal vascular structures, including the superior vena cava (SVC), aortic arch and arch vessels. The lesion caused luminal narrowing of the proximal segments of both the brachiocephalic veins near their confluence and distal segment of the SVC. No significant luminal stenosis of the aortic arch or its branches was apparent (Figures 1, 2 and 3). MRI of the thorax revealed the hypointense nature of the mass on both the *T*_1_ and *T*_2_ weighted images that suggested the possibility of fibrotic or highly cellular aetiologies (Figure 4). Based on the above imaging findings, we suggested the possibility of mediastinal fibrosis and lymphoma. Subsequently, the patient underwent mediastinal biopsy to establish the diagnosis that revealed the presence of fibrosis, polymorphic inflammatory infiltrates and phlebitis, which was consistent with the diagnosis of fibrosing mediastinitis (FM).

After confirmation of the diagnosis, the patient was started on immunosuppressive therapy consisting of oral low-dose methotrexate (10 mg week^–1^) and methylprednisolone (40 mg day^–1^) for 4 months. The methylprednisolone was then gradually tapered (10 mg day^–1^) over the next 2 months and continued for a period of 8 months. Thereafter, the patient was clinically followed-up monthly for the first 3 months and then once every 3 months for a year. A CECT scan of the chest was performed at the end of 1-year treatment period. There was gradual improvement in the clinical status of the patient during the course of the treatment. A CECT scan of the chest revealed resolution of the venous stenosis and the size of the mass after 1 year of therapy (Figure 3).

## Discussion

FM, which is also known as mediastinal fibrosis or sclerosing mediastinitis, is an uncommon, benign and progressive condition characterized by an invasive proliferation of the fibrous tissue within the mediastinum. It frequently presents with features of progressive compression of vital mediastinal structures, particularly the SVC.

FM affects persons aged between 13 and 65 years, with a strong predilection for young females.^1^ After an extensive search in the published literature, we were able to find only anecdotal data about the incidence of FM, with no definite mention of the exact prevalence.

FM has two major subtypes, granulomatous and non-granulomatous. Tuberculosis and histoplasmosis are the major causes of the granulomatous variety, while non-granulomatous FM is an idiopathic reaction to autoimmune syndromes, drugs (*e.g*. methysergide) and radiation. It is frequently associated with other fibrosing conditions such as retroperitoneal fibrosis, primary sclerosing cholangitis, orbital pseudotumours, etc.^2^

The majority of patients present with symptoms resulting from compression of the mediastinal bronchovascular structures such as progressive breathlessness, puffiness of the face, suffusion of the conjunctiva, headache and giddiness, etc.^3^

A chest radiograph may show non-specific findings that include widening of the mediastinum and enlarged lymph nodes in the subcarinal, hilar (unilateral or bilateral) and paratracheal regions, which may sometimes cause narrowing of the trachea and bronchi. Calcification of the mediastinal and hilar nodes is commonly noted in cases of the granulomatous variety.^4^

CECT scan is a useful modality that can diagnose, assess the extent and the severity of involvement. Multiplanar reformatted views are extremely helpful in depicting the degree and length of stenosis of the trachea and the mediastinal vessels. Similarly, CT angiography can be performed to reliably demonstrate the degree and extent of vascular involvement.

Two distinct radiological subtypes are seen. One has a focal, localized mass of soft-tissue attenuation with frequent occurrence of dense or stippled calcification. This pattern of involvement is frequently seen in the granulomatous variety. The other pattern has a diffuse and infiltrative nature, leading to soft-tissue masses throughout the mediastinum involving multiple compartments; calcification is not seen in this variety. The latter pattern is frequently observed in the idiopathic non-granulomatous variety. An extension of the fibrosis causing severe narrowing of the SVC can lead to SVC syndrome, with congestion of neck veins and shunting of blood into the collateral vessels around the oesophagus, stomach (downhill varices) and paravertebral region. On post-contrast study, the lesion shows variable heterogeneous enhancement.^5^

This fibrotic process can cause encasement of the main and/or branch pulmonary arteries, leading to regional or diffuse oligaemia. Pulmonary venous involvement can cause pulmonary congestion and oedema, which manifests as septal and bronchial wall thickening.^6,7^

Less commonly, it can cause constrictive pericarditis, constriction of the coronary arteries, aorta and its branches. Compression of recurrent laryngeal and/or phrenic nerves can result in neurological symptoms.

An MRI is equally useful in showing mediastinal and hilar lymphadenopathy. On *T*_1_ weighted images, the mass lesion shows intermediate signal intensity, whereas on *T*_2_ weighted images, it shows variable signal intensity with areas of high and low signal. Similar to CT, contrast enhancement is variable and heterogeneous. Foci that appear hypointense on both *T*_1_ and *T*_2_ weighted images represent calcification or dense fibrotic scar, while hyperintense areas on *T*_2_ weighted images indicate more active inflammation. Because MRI does not reliably show calcification, CT is the considered better in suspected granulomatous FM. An 18-fludeoxyglucose (FDG)-positron emission tomography scan may show evidence of radiotracer uptake in active inflammatory areas; however, owing to variable FDG avidity, it is not routinely used for the evaluation of granulomatous FM.^8,9^

Thus, we conclude that FM must be considered in the list of differential diagnosis of mediastinal lesions in a select group of patients in order to achieve early diagnosis and guide appropriate, timely therapy. This case report also highlights the importance of various imaging modalities in the diagnosis, evaluation and follow-up of patients of mediastinal fibrosis.

## Learning points

1. FM is an uncommon, benign but progressive condition characterized by an invasive proliferation of the fibrous tissue within the mediastinum that results in progressive narrowing of the bronchovascular structures.
2. The two distinct radiological subtypes of FM are focal and diffuse. The focal type presents as a localized mass with soft-tissue attenuation and stippled or dense calcification. The diffuse type manifests as a diffuse, infiltrating and homogeneous soft-tissue process throughout the mediastinum.
3. FM should be considered in the differential diagnosis when a young patient presents with symptoms of bronchovascular compression such as progressive breathlessness, chest pain, puffiness of the face, suffusion of the conjunctiva, headache and giddiness, etc.
4. CT scan and MRI play a vital role in the diagnosis, work-up and follow-up of this condition.
